# Dietary supplementation with spray-dried porcine plasma has prebiotic effects on gut microbiota in mice

**DOI:** 10.1038/s41598-020-59756-z

**Published:** 2020-02-19

**Authors:** M. Moretó, L. Miró, C. Amat, J. Polo, C. Manichanh, A. Pérez-Bosque

**Affiliations:** 1 Departament de Bioquímica i Fisiologia (Secció de Fisiologia), Facultat de Farmàcia i Ciències de l’Alimentació, Universitat de Barcelona (UB) and Institut de Nutrició i Seguretat Alimentària de la Universitat de Barcelona (INSA·UB), Barcelona, Spain; 2 APC-Europe SLU, Granollers, Spain; 3 Fundació Hospital Universitari Vall d’Hebron-Institut de Recerca, Barcelona, Spain, and CIBERehd, Instituto de Salud Carlos III, Madrid, Spain

**Keywords:** Cellular microbiology, Microbiome

## Abstract

In animal models of inflammation and in farm animals, dietary inclusion of spray-dried porcine plasma (SDP) reduces mucosal inflammation. Here, we study whether these effects could be mediated by changes in the intestinal microbiota and if these changes are similar to those induced by oral antibiotics. Weaned 21-day-old C57BL/6 mice were divided into 3 groups: the CTL group, fed the control diet; the COL group, administered low doses of neomycin and colistin; and the SDP group, supplemented with 8% SDP. After 14 days, analysis of the fecal microbiome showed that the microbiota profiles induced by SDP and the antibiotics were very different, thus, SDP has prebiotic rather than antibiotic effects. At the phylum level, SDP stimulated the presence of Firmicutes, considerably increasing the lactobacilli population. It also enhanced the growth of species involved in regulatory T-lymphocyte homeostasis and restoration of the mucosal barrier, as well as species negatively correlated with expression of pro-inflammatory cytokines. At the mucosal level, expression of toll-like receptors *Tlr2*, *Tlr4* and *Tlr9*, and mucous-related genes *Muc2* and *Tff3* with regulatory and barrier stability functions, were increased. SDP also increased expression of *Il-10* and *Tgf-β*, as well as markers of macrophages and dendritic cells eventually promoting an immune-tolerant environment.

## Introduction

Dietary plasma supplements obtained from porcine and bovine sources enhance growth in several animal species^1–4^. Such supplements are commonly used in animal husbandry because they reduce morbidity and mortality via mechanisms that involve activation of the immune system, with a special role for gut-associated lymphoid tissue (GALT) and regulation of mucosal barrier functions^5^. Studies in humans provide evidence that this kind of supplement can also improve the nutritional status and gastrointestinal symptoms in patients with enteropathy^6,7^.

The anti-inflammatory properties of spray-dried porcine plasma (SDP) have been studied in several rodent models, specifically, the model of mild intestinal inflammation induced by systemic administration of *S. aureus* enterotoxin B^8^, the model of acute lung inflammation induced by lipopolysaccharide inhalation^9,10^, the model of uterine mucosal inflammation induced by transport stress^11^, and a colitis model using knockout mice lacking the mdr1a gene that codifies for P-glycoprotein^12,13^. In all cases, the mucosal responses to the challenges showed a common pattern characterized by activation of mucosal lymphocyte populations, increasing the Tact/Treg ratio, and the secretion of pro-inflammatory cytokines. However, when animals were supplemented with 2%–8% SDP, these changes in the Tact/Treg ratio were prevented, the production of pro-inflammatory cytokines reduced and the secretion of anti-inflammatory cytokines increased^8–12,14,15^. This indicates that SDP can modulate the magnitude of inflammatory responses.

Moreover, the anti-inflammatory effects of SDP are observed if it is administered before, during or after the challenge. In models of acute inflammation, SDP was given before the challenge and the results therefore suggest that the supplement modulates receptors and regulatory pathways involved in the GALT immune responses, thereby promoting a tolerogenic profile that reduces the magnitude of the response. However, SDP is also effective with protocols that start feeding once the inflammation process has begun (as in the case of the colitis model) or even when the inflammatory response is fully established, as with the model of stress-induced mucosal uterine inflammation. This means that SDP is capable of modulating GALT both before (preventive effect) and during (therapeutic effect) inflammatory syndrome.

The first step in the anti-inflammatory cascade must take place at gut inductive sites, where SDP modulates GALT resulting in the generation of the appropriate immune responses that then spreads via the lymphatic and circulatory systems to distant mucosal lymphoid tissues such as the respiratory and genito-urinary tracts, as well as the gastrointestinal tract itself, behaving as effector sites^16^. The mechanism by which SDP modulates GALT at the inductive sites is not fully understood. The signals initiating the regulatory mechanisms may be functional SDP components, already present in the supplement or generated by its gastrointestinal digestion. This latter possibility has been shown to be the case for milk components^17^. Alternatively, they may be functional immunoglobulins in SDP, binding to luminal antigens and hence reducing the activity of luminal inflammatory stimuli, as suggested by Petschow *et al*.^3^ and Pérez-Bosque *et al*.^5^. Another possibility is that SDP modulates the intestinal commensal microbiota to promote probiotic species that stimulate cell receptors at inductive sites and eventually regulate mucosal lymphoid responses at the effector sites.

Some evidence suggests that SDP supplementation can modify the composition of the intestinal microbiota. In pigs, SDP stimulates the growth of lactobacilli in the ileum and cecum^18^, and decreases *E. coli* colonies in the small intestine^19^. Moreover, Che *et al*.^20^ have observed, in fecal samples of weaned piglets, that SDP reduces the abundance of Proteobacteria and increases Firmicutes, tripling the population of lactobacilli. In rats, SDP increased the presence of several species of *Lactobacillus* in the cecum^21^ while ovine serum immunoglobulins enriched lactobacilli and depleted enterobacteria^22^. Finally, Asmuth *et al*.^23^ reported that dietary bovine serum proteins decreased the population of fecal pro-inflammatory gamma-Proteobacteria in patients with enteropathy. All these results support the hypothesis that animal plasma supplements change the composition of the microbiota towards a probiotic profile with anti-inflammatory properties.

Since SDP is effective against bacterial toxins and viruses and protects against *E. coli*-induced inflammation in pigs^24,25^, its use has been suggested as an alternative to antibiotics^26^. Administration of low-dose antibiotics as growth promoters was a common practice in the animal farming industry^2^, and it is well documented that these practices alter intestinal microbiota and affect immune homeostasis^27^. To determine if the effects of SDP on microbiota composition and immune responses were compatible with those attributed to antibiotics, we also included an experimental group for comparing the effects of SDP supplementation with those of an antibiotic preparation containing neomycin and colistin, a combination previously widely used in farming as a growth promoter^18^.

The colonic microbiota is essential for immune homeostasis and participates in microbiota–mucosal crosstalk, cell–cell regulatory interactions, and the production of regulatory metabolites with mucosal and peripheral functions^28^. Here, we analyzed the composition of fecal microbiota, as an indicator of colonic microbiota composition, and, specifically in the colon mucosa, the levels of regulators of mucosal homeostasis and immune markers of inflammation. We also studied the expression of receptors directly involved in the regulatory mechanisms of probiotics such as membrane toll-like receptors (TLRs) and cytosolic nucleotide-binding oligomerization domain-containing protein (NOD) receptors. They are expressed in a wide variety of cell types, including intestinal epithelial enterocytes, subepithelial myofibroblasts and immune cell subsets, such as macrophages and dendritic cells^29^.

The experimental design was based on previous studies showing that animals supplemented with SDP for 14 days, starting at weaning, and then challenged with *S. aureus* Enterotoxin B^8^ or lipopolysaccharide^9^, had reduced intestinal and lung inflammatory responses, respectively. Therefore, the present study tested the hypothesis that SDP exerts preventive effects by changing the microbiota composition and consequently modulating the mucosal immune mechanisms toward a tolerogenic profile.

## Results

### Effects of antibiotics on microbiota composition

**Figure 1:**
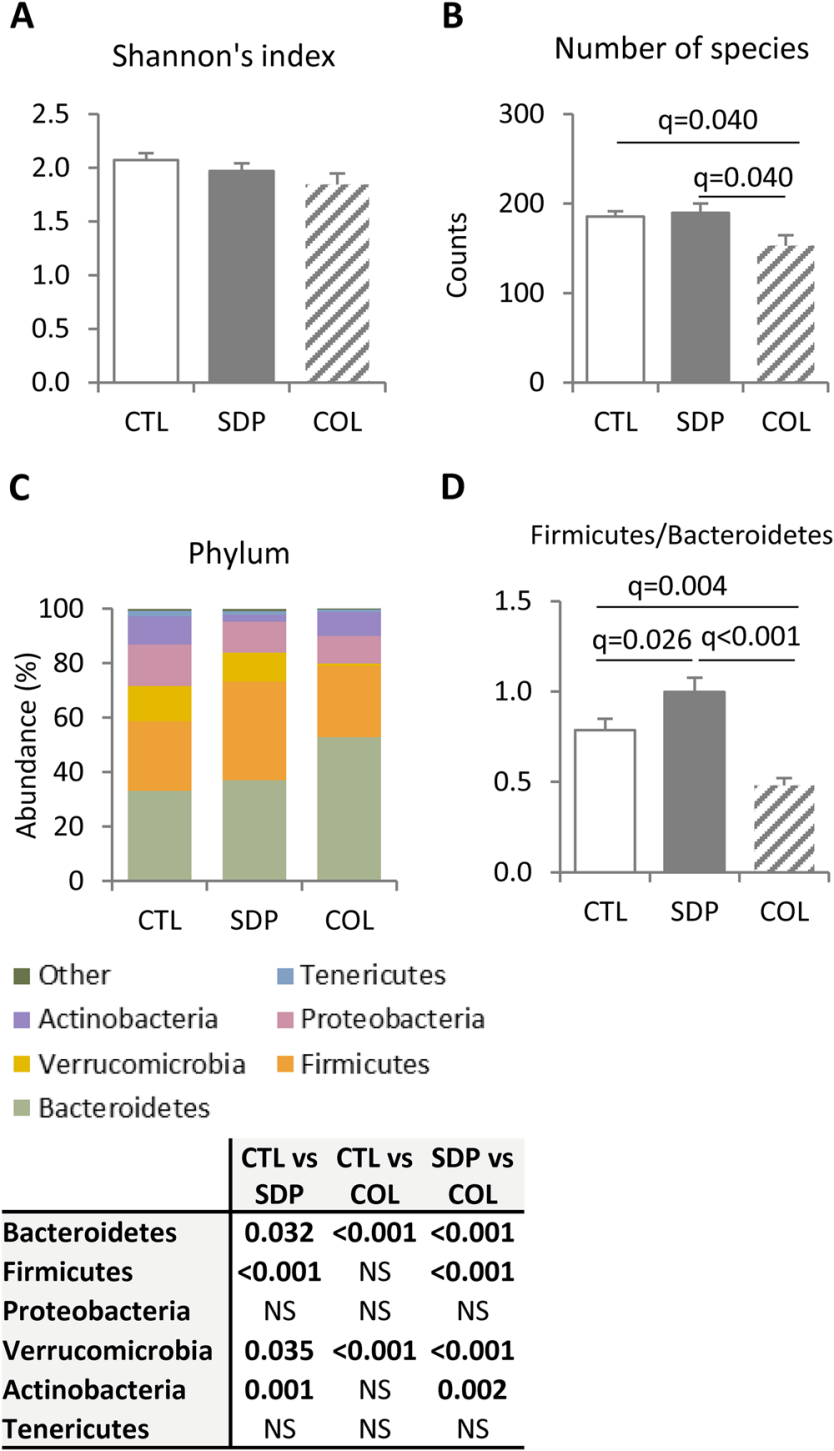
Microbial Shannon’s index (**A**), number of species (**B**), bacterial composition at phylum level (**C**) and ratio between Firmicutes and Bacteroidetes (**D**) in fecal microbiota. Samples were collected from mice fed a control diet (CTL) or a diet supplemented with 8% spray-dried porcine plasma (SDP) and mice fed control diet and treated with Coliphur (COL; daily dose: 25 mg/kg neomycin and 10 mg/kg colistin) for 14 days. Results are expressed as mean ± SEM (n = 9–10 mice). Statistical differences were considered significant at q < 0.05 (corrected p values).

Since SDP is used as an alternative to antibiotics^26^, we analyzed the extent to which its effects on microbiota composition are comparable to those induced by the low doses of antibiotics. Figure 1A shows that neither SDP nor the neomycin/colistin preparation affected the Shannon (diversity) index; however, antibiotics reduced the total number of species (Fig. 1B) while SDP did not. At the phylum level (Fig. 1C), the effects on microbiota composition were very different as antibiotics enhanced the Bacteroidetes population and induced a dramatic reduction of Verrucobacteria; while these effects were not observed in the animals fed SDP (both q < 0.001). Moreover, SDP increased the Firmicutes-to-Bacteroidetes ratio, while antibiotic treatment reduced it (Fig. 1D). The effects of the antibiotic combination on families, genera, and species are shown in Supplementary Table 3 and Supplementary Fig. 1.

### Effects of dietary supplementation with SDP on fecal microbiota composition

**Figure 2:**
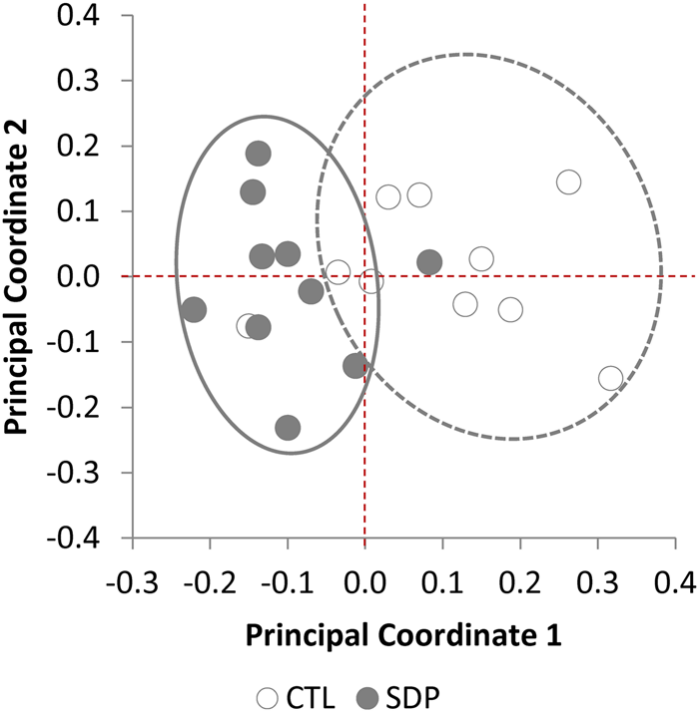
Principal coordinate analysis (PCoA) plot of Illumina sequence data at family level, from fecal bacterial sequences from mice fed a control diet (CTL) or a diet supplemented with 8% spray-dried porcine plasma (SDP) for 14 days. The x- and y-axes are indicated by the first and second coordinates, respectively (n = 10 mice).

In the CTL group, the dominant phyla were Bacteroidetes (33 ± 1.7%) followed by Firmicutes (25 ± 1.7%), Verrucobacteria (16 ± 1.5%), Proteobacteria (13 ± 1.5%) and Actinobacteria (10 ± 2.1%), with the remaining 3 ± 0.5% of the bacterial population dominated by the phylum Tenericutes (Fig. 1C). Dietary SDP increased the relative amount of Firmicutes up to 36.3 ± 2.0% (q < 0.001) and Bacteroidetes up to 37.1 ± 1.4% (q = 0.032), and this increased the Firmicutes-to-Bacteroidetes ratio from 0.77 to 0.98 (q = 0.026). Meanwhile, SDP decreased the population of Verrucobacteria to 11.3 ± 1.2% (q = 0.035) and Actinobacteria to only 2.6 ± 1.0% (q < 0.001). The principal coordinate analysis (PCoA) defined two different populations, as shown for the family level in Fig. 2; with the same finding repeated at other taxonomic levels.

**Figure 3:**
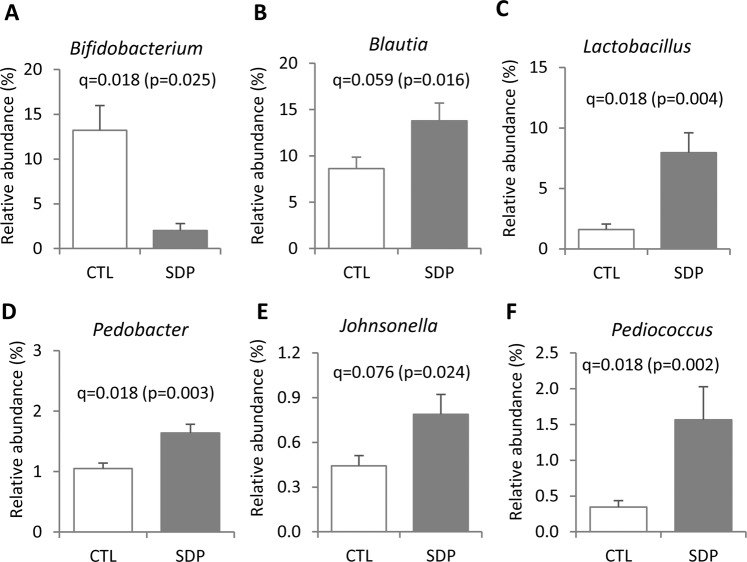
Effects of SDP on fecal microbial composition at genus level. Mice were fed a control diet (CTL) or a diet supplemented with 8% spray-dried porcine plasma (SDP) for 14 days. Results show the mean relative abundance ± SEM (n = 9–10 mice). Statistical differences were considered significant at q < 0.05 (corrected p values).

**Figure 4:**
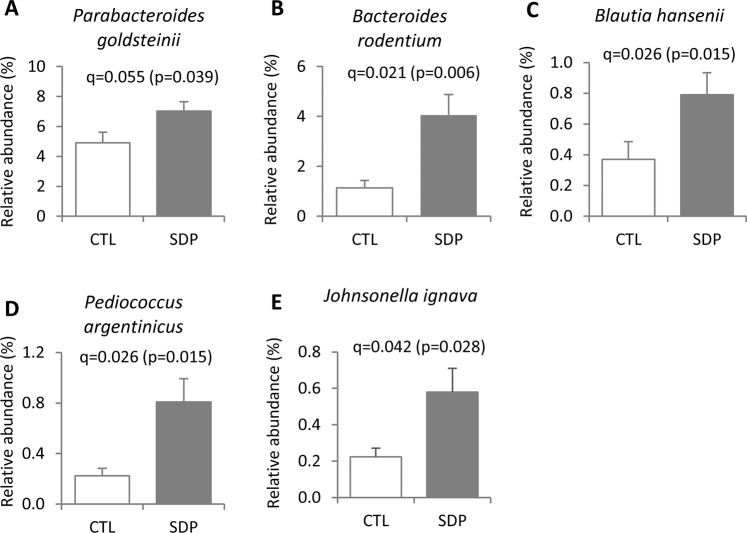
Effects of SDP on fecal microbial composition at species level. Mice were fed a control diet (CTL) or a diet supplemented with 8% spray-dried porcine plasma (SDP) for 14 days. Results are means ± SEM (n = 9–10 mice). Only significant effects on bacterial phylotypes that had >0.1% of the total sequence reads are shown. Statistical differences were considered significant at q < 0.05 (corrected p values).

**Figure 5:**
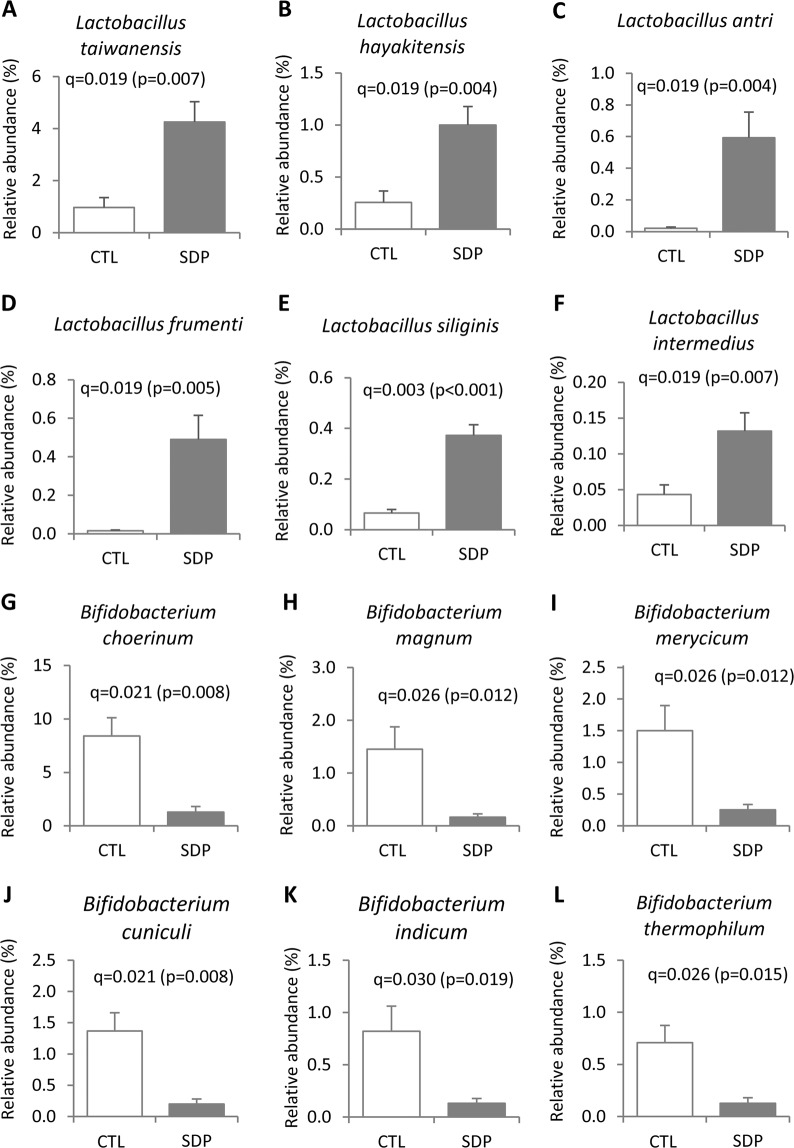
Effects of SDP on fecal species of the genus *Lactobacillus* and *Bifidobacterium*. Mice were fed a control diet (CTL) or a diet supplemented with spray-dried porcine plasma (SDP) for 14 days. The figure shows all species that were modified by SDP feeding. Results are expressed as mean ± SEM (n = 9–10 mice). Statistical differences were considered significant at q < 0.05 (corrected p values).

The effects of SDP supplementation at the family level are shown in Table 1. SDP increased Porphyriomonadaceae (q = 0.095) from the Bacteroidetes phylum, and Lactobacillaceae (q = 0.049) from the Firmicutes phylum. However, it considerably decreased the Bifidobacteriaceae population (q = 0.041) from the phylum Actinobacteria. Figure 3 shows the effects of SDP on the genera that are relevant from the functional point of view: five of them (*Blautia, Lactobacillus, Pedobacter, Johnsonella and Pediococcus*) were significantly stimulated, while *Bifidobacterium* was markedly inhibited (all q < 0.02, except *Blautia* and *Johnsonella* with q = 0.059 and q = 0.076, respectively). Figures 4 and 5 show the bacterial species whose growth was affected by SDP supplementation.

**Table 1:** Bacterial composition of the fecal microbiota at family level.

Phylum	Family	CTL^a^ (%)	SDP (%)	q
Bacteroidetes	Bacteroidaceae	15.3	10.7	NS
	Porphyromonadaceae	6.42	8.73	**0.095**
	Sphingobacteriaceae	4.15	5.04	NS
	Flavobacteriaceae	3.63	4.13	NS
	Odoribacteriaceae	0.48	0.77	NS
	Prevotellaceae	0.36	0.08	NS
Firmicutes	Lachnospiraceae	12.1	16.8	NS
	Clostridiaceae	5.18	5.70	NS
	Lactobacillaceae	2.61	11.4	**0.049**
	Erysipelotrichaceae	1.94	2.62	NS
	Ruminococcaceae	2.88	4.08	NS
	Eubacteriaceae	0.45	0.83	NS
Proteobacteria	Alcaligenaceae	0.96	0.46	NS
	Desulfovibrionaceae	0.87	0.56	NS
Verrucomicrobia	Verrucomicrobiaceae	19.0	14.5	NS
Actinobacteria	Bifidobacteriaceae	12.4	1.83	**0.041**
Tenericutes	Entomoplasmataceae	2.05	1.10	NS
Other	Other (up to 129)	9.22	10.7	—

^a^Mice fed a control diet (CTL) or a diet supplemented with spray-dried porcine plasma (SDP) for 14 days. Results are expressed as percent of the total population at family level (mean ± SEM, n = 9–10 mice). Statistical differences were considered significant at q < 0.05 (corrected p values).

### Effects of dietary SDP on colonic mucosal receptors and immune regulators

**Figure 6:**
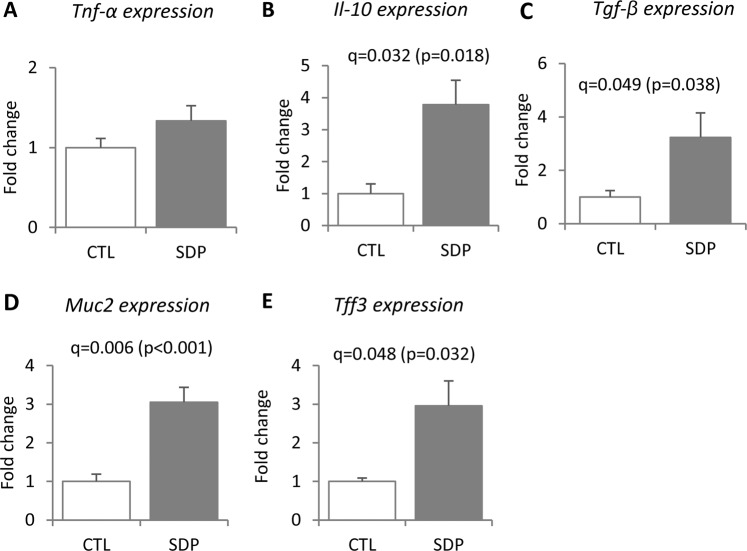
Effects of SDP on *Tnf-α* (**A**)*, Il-10* (**B**), *Tgfβ* (**C**), *Muc2* (**D**) and *Tff3* (**E**) expression in the colon mucosa. Mice were fed a control diet (CTL) or a diet supplemented with 8% spray-dried porcine plasma (SDP) for 14 days. Results are expressed as mean ± SEM (n = 5–6 mice). Statistical differences were considered significant at q < 0.05 (corrected p values).

The study of the expression of mucosal cytokines showed that SDP supplementation for 14 days stimulated the expression of the anti-inflammatory cytokines *Il-10* and *Tgf-β* (q = 0.032 and q = 0.049, respectively); while expression of the pro-inflammatory *Tnf-α* was not affected (Fig. 6). Dietary SDP did not modify the abundance of the epithelial adhesion molecule E-cadherin or the junctional protein occludin (Supplementary Fig. 2) but did increase those of the goblet cell secretory products *Muc2* and *Tff3*, which are involved in the regulation of mucosal barrier stability and permeability (q = 0.006 and q = 0.048, respectively).

**Figure 7:**
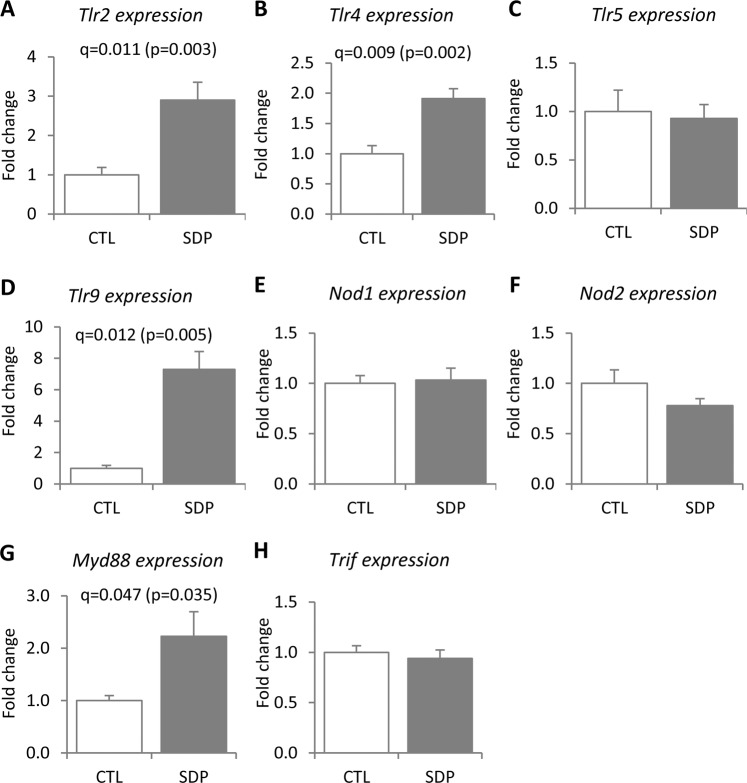
Effects of SDP on *Tlr2* (**A**), *Tlr4* (**B**), *Tlr5* (**C**), *Tlr9* (**D**)*, Nod1* (**E**)*, Nod2* (**F**)*, Myd88* (**G**) and *Trif* (**H**) expression in the colon mucosa. Mice were fed a control diet (CTL) or a diet supplemented with 8% spray-dried porcine plasma (SDP) for 14 days. Results are expressed as mean ± SEM (n = 5–6 mice). Statistical differences were considered significant at q < 0.05 (corrected p values).

We analyzed some mucosal receptors with relevant roles in microbiota–mucosa crosstalk (Fig. 7A–D). SDP stimulated the expression of the membrane receptors *Tlr2*, *Tlr4*, and *Tlr9* by nearly 3-fold, 2-fold, and more than 7-fold (q = 0.011, q = 0.009, and q = 0.012, respectively) but had no effect on *Tlr5*. Cytosolic NOD1 and NOD2 receptors, which can also respond to bacterial components, were not affected by SDP (Fig. 7E,F). Finally, we studied adaptor proteins recruited by TRLs to initiate signal transduction pathways and regulate cytokine expression. The myeloid cell-specific gene *Myd88* was significantly increased in the SDP-supplemented mice, whereas TIR domain-containing adapter-inducing interferon-β (*Trif*) was not (Fig. 7G,H).

**Figure 8:**
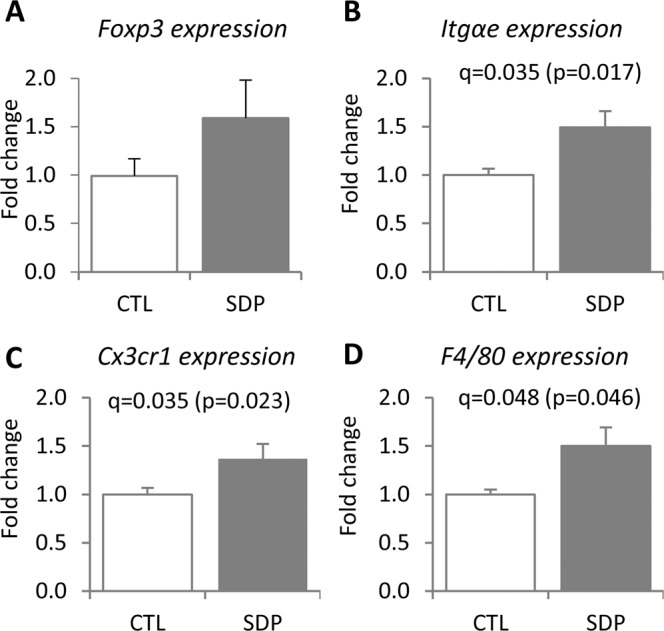
Effects of SDP on *Foxp3* (**A**)*, Itgαe* (**B**), *Cx3cr1* (**C**) and *F4/80* (**D**) expression in the colon mucosa. Mice were fed a control diet (CTL) or a diet supplemented with 8% spray-dried porcine plasma (SDP) for 14 days. Results are expressed as mean ± SEM (n = 5–6 mice). Statistical differences were considered significant at q < 0.05 (corrected p values).

Because the lymphoid population of the colon mucosa contains different types of cells that may participate in tolerogenic responses, we analyzed some markers that are specific for macrophages (F4/80 and Cx3cr1) and dendritic cells (integrin α_E_). SDP stimulated both populations (all q < 0.005, Fig. 8). The expression of Foxp3, a specific marker of natural T-regulatory cells, was not stimulated by SDP under basal conditions.

## Discussion

The present study shows that feeding mice with SDP changes the microbiota composition at several taxonomic levels, enhancing probiotic families and species that regulate mucosal barrier permeability and promote mucosal tolerance. The changes in the microbiota profile induced by SDP differ markedly from those resulting from the administration of low doses of antibiotics used in farm animals to promote growth^18^. Administration of low-dose antibiotics alters the intestinal microbiota and affects immune homeostasis, particularly in early life, because the changes induced in the microbiota may lead to the loss of species with important functions, increasing the risk of several diseases in adulthood^30^. Thus, whereas antibiotics reduced the total number of bacterial species, SDP did not; at the phylum level, antibiotics reduced Verrucobacteria 16-fold, while SDP had no effect; at the family level, SDP stimulated lactobacilli and inhibited bifidobacteria and these effects were not reproduced by antibiotics. Further evidence to distinguish the effects of SDP on the microbiota from those of antibiotics is that the latter can affect the responsiveness to vaccines. For example, pigs administered cephalosporin have a reduced immune response to vaccination against the influenza A virus^31^ while early-life exposure of mice to antibiotics shapes their subsequent responses to vaccines^32^. Dietary SDP promotes a microbiota profile with probiotic properties that may even improve the response to vaccination, as shown in aged SAMP8 mice after nasal vaccination with *S. aureus* enterotoxin B^33^. These results lead us to conclude that the anti-inflammatory and growth-promoting effects of SDP rely on microbial changes unrelated to alleged “antibiotic-like” properties of the supplement.

Altogether, our results demonstrate that 14 days of SDP supplementation suffices to induce profound changes in the gut microbiota. There is a significant increase in phyla Firmicutes and Bacteroidetes both producers of short-chain fatty acids (SCFAs). Acetate, propionate and butyrate are essential for the maintenance of mucosal immunity and mucosal barrier function^34^, and they promote tolerance through GALT modulation^35^. SDP increases *Parabacteroides goldsteinii*, a species in the Porphyromonadaceae family that has anti-inflammatory effects *in vivo*, reducing *Tnf-α* expression in the liver^36^ while inducing *Il-10* expression in the proximal colon of mice^37^. The genus *Blautia* (Lachnospiraceae family) is positively correlated with low inflammatory status and high cognition scores^38^, and these results are compatible with the effects of SDP reported on systemic inflammation^39^ and cognitive functions^40^.

SDP supplementation had notable effects on probiotic species. The intestine houses numerous lactic acid-producing bacteria that promote health by producing metabolites that can interact with the host’s own metabolism and immune system^41^. Most probiotics belonging to the genera *Lactobacillus* and *Bifidobacterium* modulate the GALT and prevent adhesion of and colonization by pathogens^42^. SDP strongly stimulated the growth of members of the Lactobacillaceae family, indicating that SDP has prebiotic effects. The abundance of Lactobacillaceae increased 3-fold and that of the genus *Lactobacillus* increased 5-fold. These observations confirm previous results showing that SDP enhances the frequency of detection of *Lactobacillus* species in the rat cecum^21^ as well as in pig ileum^18^ and pig colon^20^.

The effects of SDP that promote the growth of commensal species from the Lactobacillaceae family while reducing the presence of *Bifidobacterium* strains may not affect the number of lactic acid-producing bacteria, as the total number of microbial species in feces is not affected by SDP. This suggests that the probiotic metabolic profile of the microbiota is not modified. Interestingly, though most prebiotics stimulate growth of both lactobacilli and bifidobacteria, SDP specifically simulates the proliferation of lactobacilli at the expense of bifidobacteria. Perhaps this is due to SDP composition being markedly different from that of conventional prebiotics, as it is mostly composed of peptides and proteins. This means that while some may be digested and absorbed in the small intestine, others resist digestion and are excreted in feces^43^. In the rat intestine, a soy milk supplement increased *Lactobacillus* and decreased *Bifidobacterium* strains, similarly to what was observed in the present study^44^. The distinct effects of SDP can be attributed to the differences in bacterial substrate preferences. Bottari *et al*.^45^ demonstrated that bifidobacteria preferred peptides with 4 or 5 residues, while lactobacilli mostly consume dipeptides. Hence, the peptide composition of SDP, and the structure of the peptides resulting from digestion of its protein components, might explain the different growth of commensal lactobacilli and bifidobacteria.

The levels of *L. taiwanensis* and *L. frumenti* were increased several-fold by SDP; and *L. antri*, although present in only low proportion, was increased 60-fold. It is worth noting that *L. antri and L. frumenti*, are representative strains within the *L. reuteri* subgroup of lactobacilli with well-known probiotic properties^46^. *L. taiwanensis* has regulatory functions and interacts with dendritic cells of the lamina propria, as part of the bacterial-GALT crosstalk that maintains mucosal homeostasis^47^ and these effects are consistent with the increased expression of the dendritic cell marker integrin α_E_ observed in the present study. Mice exposed to *L. taiwanensis* show increased frequency of Treg cells in both mesenteric lymph nodes and Peyer’s patches^48^, that then reside in the gut, where they expand and produce a tolerant environment for specific antigens^49^. These observations agree with our results showing that SDP supplementation consistently increases the percentage of Treg in acute models of mucosal inflammation^9,50^ as well as in aging^39^ and colitis^13^ mice models. SDP also stimulates the expression of *Muc2*, a protein that, in addition to its role in the formation of the mucous barrier, signals pathways that regulate dendritic cells of the lamina propria, thus enhancing oral tolerance^51^.

*Blautia* has also been shown to regulate the expression of tight-junction ZO-1 and maintenance of mucosal permeability^52^ and *L. frumenti* is a commensal species that, when given by oral gavage to piglets prior to weaning, decreases the relative abundance of opportunistic pathogens and improves intestinal mucosal integrity via a mechanism involving up-regulation of ZO-1, occludin, and claudin-1. We have previously shown that inflammation reduces the expression of the tight junction protein ZO-1 and adherent junction protein β-catenin in the small intestine and that SDP can prevent this decrease^50,53^. Because *Blautia* was increased 1.4-fold by SDP, we hypothesized that the effects of SDP on mucosal integrity might be mediated by this species. However, there were no changes in the levels of occludin and E-cadherin, as representative proteins of the tight junction and adherens junction, respectively, suggesting that SDP cannot affect permeability in unchallenged animals.

In the colon mucosa, dietary SDP induces Tgf-β expression, which in turn upregulates the expression of integrin α_E_ in mucosal immune cells^54^. Among the cells expressing this marker are dendritic cells required for the activation of FoxP3 regulatory T cells to induce tolerogenic responses; this is important in the large intestine because this region is exposed to commensal bacteria driving inflammation^55^. We have also shown that SDP stimulates the expression of F4/80 and CX3CR1, which are macrophage markers in the gut with important roles in the priming of immune responses. CX3CR1+ macrophages produce immunoregulatory cytokines such as IL-10 and can also facilitate the differentiation and maintenance of Treg within the lamina propria^56^. They are also required for the induction of the efferent CD8+ reg-T cells required for peripheral tolerance^57^.

Mucosal barrier functions depend on the permeability properties of the epithelial cell lining. These in turn result from a combination of the chemical barrier composed of antimicrobial peptides (AMPs) secreted by the epithelium to control bacterial growth, and the physical barrier made of mucus, mainly secreted by goblet cells. Inflammation reduces the expression of AMPs and the secretion of mucous components such as *Muc2* or the barrier stability component *Tff3*, both secreted by goblet cells^58^. Oral lactobacilli stimulate intestinal antimicrobial activity *in vivo* and increase Muc2 secretion^59^, indicating that probiotics can regulate epithelial function providing mucosal protection. SDP reinforces mucosal barrier stability and stimulates epithelial regeneration, as both *Muc2* and *Tff3* minimize the consequences of inflammation resulting from the challenges (and preserve these important functions). These results are also in keeping with previous observations in the mdr1−/− mouse model of colitis, showing that SDP can increase the number of goblet cells in the colon mucosa^60^. Our current results, showing that SDP stimulates lactobacilli proliferation, further support the view that the regulatory effects of SDP on mucosal barrier functions are mediated by changes in microbiota composition.

The microbiota interacts with epithelial and lamina propria cells through TLRs that are transmembrane receptors and cytosolic NOD receptors, with the capacity to distinguish between pathogen and commensal microbes. Our results show that TLRs rather than NOD receptors are involved in the tolerogenic responses induced by SDP. It is well known that, once stimulated, TLRs activate intracellular signal pathways mediated by MAP kinases and NF-κB that eventually trigger pro-inflammatory immune responses^61^. However, TLRs also modulate transduction pathways that induce anti-inflammatory responses. We hypothesized that SDP might modulate inflammation and barrier function by regulation of mucosal TLR expression in the colon mucosa. Our results show that *Tlr2*, *Tlr4* and *Tlr9* were indeed overexpressed after the 14-day SDP supplementation period, coinciding with several-fold stimulation of lactobacilli and other SCFA-producing families such as Lactobacillaceae and Porhiromonadaceae. Castillo *et al*.^62^ observed that the administration of probiotic *L. casei* to healthy mice for seven days increased the expression of *Tlr*2, *Tlr4* and *Tlr9* in the small intestine, which suggests that our results for TLRs may also be mediated by lactobacilli induced by SDP.

SDP stimulates *Tlr2* expression and expression of both *Il-10* and *Tgf-β*, consistent with previous observations in healthy unchallenged rodents^53^, and these two variables may be correlated. The capacity of *Tlr2* to induce pro- or anti-inflammatory responses depends on whether it dimerizes with other receptors. Hence *Tlr2-Tlr1* heterodimers induce anti-inflammatory responses mediated by *Il-10* in human small intestine^63^ and in antigen-presenting cells from porcine Peyer’s patches incubated with lactobacilli^64^. *Tlr2* stimulates the internalization of lactobacilli, as a pathway for the activation of Treg lymphocytes, and increases the number of mucosal tolerogenic dendritic cells, which prime Treg cells and the production of anti-inflammatory cytokines^65^.

Kaji *et al*.^66^ have shown that bacterial ligands for *Tlr2, Tlr4, and Tlr9* may convert the cytokine production pattern from predominantly pro-inflammatory to anti-inflammatory, indicating that probiotic induction of pro- and anti-inflammatory cytokines can be modified by co-stimulation with microbial components; an effect that has also been observed in monocytes^67^ and in dendritic cells^68^. The kind of response (either pro- or anti-inflammatory) further depends on TLR compartmentalization in the cell. For example, *Tlr4* expressed on the cellular membranes plays a pro-inflammatory signaling role involving *MyD88* co-activation;^69^ while intracellular *Tlr4* plays an anti-inflammatory role, inducing the expression of *Il-10*^70^. *Tlr9* activation induces conventional dendritic cells to secrete anti-inflammatory *Il-10*, thus attenuating inflammatory responses and liver injury in mice^71^. In intestinal epithelial cells, *Tlr9* plays an important homeostatic role, protecting mice from experimental colitis^72^ and these results are compatible with the anti-inflammatory effects of SDP observed in *mdr1−/−* mice^13,73^.

In summary, results from the present study and from other laboratories are consistent with the hypothesis that the mechanism by which SDP modulates GALT involves overexpression of TLR at the inductive sites and stimulation of the Myd88 pathway as first steps towards regulating inflammatory responses at the effector sites as shown by others^74^.

In conclusion, this study demonstrates that animal plasma supplements can modulate the composition of microbiota and provides a mechanistic explanation: it links the bacterial families and species promoted by SDP with the expression of mucosal makers and immune regulators involved in intestinal and systemic homeostasis. SDP stimulates the growth of some probiotic species and the expression of mucosal regulatory signals and anti-inflammatory pathways. Specifically, SDP increases the presence of bacterial families that enhance the intestinal barrier function and species that are well-known mediators of anti-inflammatory and tolerogenic responses.

## Material and Methods

### Animals and diets

Male C57BL/6 mice were purchased from Envigo (Bresso, Italy) and kept under stable temperature and humidity conditions, with a 12 h light–12 h dark cycle and free access to food and water. All protocols used in the present study were approved by the Animal Experimentation Ethics Committee of the Universitat de Barcelona (Ref. 503/14), following the guidelines for the Care and Use of Laboratory Animals of the regional government (DAAM 7939, Generalitat de Catalunya, Spain). At day 21 (weaning) 30 mice were equally distributed at random in three groups; the CTL group, fed the control diet, the SDP group, supplemented with 8% SDP, and the COL group fed the control diet and administered with a mixture of antibiotics in the drinking water, as described below.

SDP is a protein-rich ingredient obtained from industrial fractionation of blood from healthy pigs. Blood is collected with an anticoagulant (sodium citrate or sodium phosphate) and centrifuged to separate the plasma fraction from blood cells. The plasma is then concentrated through membranes and spray-dried. With this procedure, proteins and peptides preserve most of their biological activity^75^. Control and SDP diets were designed to provide balanced energy and nutrients. The experimental diets were prepared by APC-Europe SLU from base ingredients provided by Envigo, and their composition is detailed in Supplementary Table 1.

### Antibiotic treatment

A group of mice fed with the control diet received a pharmaceutical preparation containing 100 mg/L neomycin and 40 mg/L colistin (Coliphur; Maymó, Spain; COL group). The estimated daily doses of antibiotics were 25 mg/kg for neomycin and 10 mg/kg for colistin. This antibiotic dose is similar to that being used in pigs^18^.

### Sample collection

Feces were collected at days 33–35 of life (12–14 days on diet) in clean conditions. Samples were immediately frozen in liquid N_2_ and maintained at −80 °C until use. At the end of the experiment, mice were anaesthetized with xylazine/ketamine killed by exsanguination. Colon samples were obtained as previously described^14^. Briefly, the colon was washed with phosphate-buffered saline, and the colon mucosa was scraped and quickly frozen at −80 °C for further analysis.

### Extraction and purification of total genomic DNA

DNA was extracted according to Santiago *et al*.^76^ with some modifications. The method is based on microbial disruption by bead-beating because it allows a better detection of Gram-positive bacteria and because it reduces the miscellaneous populations to very low values. Briefly, samples (70 mg feces/mice) were suspended in 0.25 mL of 4 M guanidine thiocyanate (Sigma Aldrich, St. Louis, MO, USA), 40 μL of 10% N-lauroyl sarcosine (Sigma Aldrich) and 0.5 mL of 5% N-lauroyl sarcosine (Sigma Aldrich). DNA extraction was carried out by mechanical disruption of the microbial cell wall using Zirconia/silica beads of 0.1 mm diameter (BioSpec Products, Bartlesville, OK, USA). The disruption was performed by shaking the mixture using the FastPrep®−24 (MP Biomedicals, Solon, OH, USA). Tubs were added with polyvinylpolypyrrolidone (15 mg, Sigma Aldrich) and then vortexed and centrifuged for 3 min at 12,000 *g*. After recovery of the supernatant, the pellet was washed with 0.5 mL of TENP (50 mM Tris [pH 8], 20 mM EDTA [pH 8], 100 mM NaCl, 1% polyvinylpolypyrrolidone) and centrifuged for 3 min at 12,000 *g*. Supernatants were transferred to new tubes and pellets were washed three times. Finally, nucleic acids were recovered from clear lysates by isopropanol precipitation. Pellets were resuspended and pooled in 0.225 mL of 100 mM phosphate buffer, pH 8, and 50 μL of 5 M potassium acetate (all reagents were from Sigma Aldrich). Tubes were placed on ice for 90 min and centrifuged at 16,000 *g* for 30 min. The supernatants were transferred to tubes containing 5 μL of RNase (4 mg/ml, Qiagen, Venlo, The Netherlands) and incubated at 37 °C for 30 min. Nucleic acids were precipitated by the addition of 50 μL of 3 M sodium acetate (Sigma Aldrich) and 1 mL of absolute ethanol (JT Baker, Deventer, The Netherlands). Tubes were then incubated for 10 min at room temperature, and nucleic acids were recovered by centrifugation at 20,000 *g* for 15 min. DNA pellets were finally washed with 70% ethanol, dried, and resuspended in 0.1 mL of water. The quantification of DNA was done using a NanoDrop ND-100 Spectrophotometer (Thermo Fisher Scientific, Waltham, MA, USA).

### 16S rDNA gene analysis

The extracted genomic DNA was processed and the variable V3 and V4 regions of the 16S rRNA gene were amplified. The primers to detect 16S rRNA gene were: 16S forward 5′-TCGTCGGCAGCGTCAGATGTGTATAAGAGACAGCCTACGGGNGGCWGCAG-3′ and reverse 5′ -GTCTCGTGGGCTCGGAGATGTGTATAAGAGACAGGACTACHVGGGTATCTAATCC-3′. High-through sequencing was done using the Illumina MiSeq platform (Illumina, San Diego, CA, USA) at the Genomics and Bioinformatics Service, Universitat Autònoma de Barcelona (Bellaterra, Spain).

### Real-time PCR analysis

Total RNA was extracted with TRIzol reagent (Life Technologies, Carlsbad, CA, USA) following the manufacturer’s instructions. RNA extraction and retrotranscription were carried out as previously described^39^. The mouse primers used are listed in Supplementary Table 2. Real-time PCR was performed using a cDNA template in a 20 µL reaction containing 0.2 μmol/L of each primer and SsoAdvanced Universal SYBR Green Supermix (Bio-Rad, Hercules, CA, USA) and carried out on a MiniOpticon Real-Time PCR System (Bio-Rad, Hercules, CA, USA). To confirm PCR amplification of the intended product, representative samples were analyzed by electrophoresis on a 4% agarose gel. Product fidelity was confirmed by melt-curve analysis. TaqMan gene expression assays (Applied Biosystems, Foster City, CA, USA) were used for the following genes: Toll-like receptor 2 (*Tlr2*, Mm00442346_m1), Toll-like receptor 4 (*Tlr4*, Mm00445273_m1), Toll-like receptor 9 (*Tlr9*, Mm00446193_m1) and Myeloid differentiation primary response 88 (*Myd88*, Mm00440338_m1) following the manufacturer’s instruction. Each PCR run included duplicates of reverse transcription for each sample and negative controls (reverse transcription-free samples, RNA-free sample). Quantification of the targeted gene transcripts was done using hypoxanthine phosphoribosyltransferase 1 (*Hprt1*, Mm00446968_m1) gene expression as reference, and was carried out with the 2^−ΔΔCT^ method^77^.

### Statistics

Hierarchical clustering and ordination of the community structures were performed using a Principal Coordinate Analysis (PCoA) plot by Illumina MiSeq analyzer (Illumina, San Diego, CA, USA). Results are presented as mean ± SEM. The statistical analysis was performed using GrapPad Prism® software v 7.01 (GraphPad Software, Inc., La Jolla, CA, USA). Grubb´s test was performed to determine outliers and the Shapiro-Wilk test was used to check the normality of data distribution. When comparing three groups (i.e. CTL, SDP and COL), the ANOVA test was used when data were normally distributed; otherwise the non-parametric Kruskal-Wallis test was carried out. When only Control and SDP conditions were compared, the Student t-test was used when data were normally distributed; otherwise, the non-parametric Mann-Whitney U-test test was used. All p-values were corrected for multiple testing using the false discovery rate (FDR) correction (Benjamini-Hochberg). Statistical differences were considered significant at q < 0.05. A q value between 0.05 and 0.1 was suggestive of a true effect^78^.

## Supplementary information

Supplementary data.
