# Supplementary material for: Dietary supplementation with spray-dried porcine plasma has prebiotic effects on gut microbiota in mice
Source: Sci Rep. 2020 Feb 19;10:2926. doi: 10.1038/s41598-020-59756-z (PMC7031359; doi:10.1038/s41598-020-59756-z)
Supplement: Supplementary file 1 — Supplementary data. [file 41598_2020_59756_MOESM1_ESM.pdf]

Supplementary data

Dietary supplementation with spray-dried porcine plasma has prebiotic effects on gut microbiota in mice

M. Moretó[1†*], L. Miró[1,2†], C. Amat[1], J. Polo[2], C. Manichanh[3], A. Pérez-Bosque[1]

1. Departament de Bioquímica i Fisiologia (Secció de Fisiologia), Facultat de Farmàcia i Ciències de l'Alimentació, Universitat de Barcelona (UB) and Institut de Nutrició i Seguretat Alimentària de la Universitat de Barcelona (INSA·UB), Barcelona, Spain.

2. APC-Europe SLU, Granollers, Spain;

3. Fundació Hospital Universitari Vall d'Hebron-Institut de Recerca, Barcelona, Spain, and CIBERehd, Instituto de Salud Carlos III, Madrid, Spain.

†These authors contributed equally to this work.

*Correspondence: Prof. Miquel Moretó (mmoreto@ub.edu), Secció de Fisiologia, Departament de Bioquímica i Fisiologia, Facultat de Farmàcia i Ciències de l'Alimentació, Av. Joan XXIII, 27-31, 08028 Barcelona, Spain. T. +34 934024505.

Short title: Spray-dried porcine plasma promotes a probiotic microbiota

Supplementary Materials & Methods

Western blot. Western blot procedures were performed according to a previous study (García-Just et al., 2019). Samples of colon mucosa were homogenized and protein concentration was determined using the Bradford method (Bio-Rad, Hercules, CA, USA). Equal amounts of protein (30 µg) were separated on 10% SDS-PAGE and transferred to polyvinylidene difluoride membranes (Bio-Rad, Hercules, CA, USA). Membranes were incubated overnight at 4°C with diluted primary antibodies against β-actin (clone AC-74; 1:80,000; Sigma Aldich, St. Louis, MO, USA), occludin (clone OC-3F10, Life Technologies, Rockford, IL, USA) and E-cadherin (clone H-108, 1:500; Santa Cruz Biotechnology, Dallas, TX, USA). Membranes were washed and incubated with horseradish peroxidase (HRP)-conjugated secondary antibodies (Sigma, St. Louis, MO, USA) for 2 h at room temperature. Protein bands were visualized using a chemiluminescence detection kit Clarity and ChemiDoc XRS+ instrument (both from Bio-Rad, Hercules, CA, USA). After their detection, hybridization bands were quantified using ImageJ gel analyzer software.

Supplementary Table 1. Diet composition.

Ingredients	Control feed	SDP[1] feed
	g/kg	g/kg
SDP	-	80
Dried skim milk	530.7	340.5
Corn starch	199.3	308.8
Sucrose	94.5	94.5
Soybean oil	70	70
Cellulose	50	50
AIN-93-G-MX (94046)[2]	35	35
AIN-93 VX (94047)[2]	15	15
Choline bitartrate	3	3
Methionine	2.5	3.2

[1]SDP (spray-dried porcine plasma) was provided by APC-Europe, SLU (Granollers, Spain); [2]AIN-93 VX, vitamin mix; AIN-93-G-MX, mineral mix, both provided by Envigo (Bresso, Italy).

Supplementary Table 2. Primers used for real-time PCR experiments

Primer	Forward (5'-3')	Reverse (5'-3')	Fragment size
Cx3cr1	ATTGGCTTCTTTGGGGGCAT	GTTGTTCATGGAGTTGGCGG	90 bp
F4/80	AACATGCAACCTGCCACAAC	TTCACAGGATTCGTCCAGGC	110 bp
Foxp3	TTCCTTCCCAGAGTTCTTCCAC	ATGGCCCATCGGATAAGGGT	93 bp
Il-10	GGCGCTGTCATCGATTTCTCCCC	TGGCCTTGTAGACACCTTGGTCTT	102 bp
Itgαe	TGACAAAGACTCAGGACCACAC	ATAGCACAGACCACTGAATGCC	106 bp
Muc2	TGCTGCTGACGAGTGGTTGGTG	CGGACGCTTGGTGGTGAGGC	76 bp
Nod1	CCTTGCCTGTGAGCAGAAAGTA	GGTATGTGCCATGCTTTGCTT	112 bp
Nod2	CAACAATGGCATCACCTACCG	TGTGTTCCCTCGAAGCCAAA	94 bp
Tff3	GCCCTCTGGCTAATGCTGTT	CTTGGAGACAGGACGCCAACGTA	80 bp
Tgf-β	CAGTGGCTGAACCAAGGAGACGG	CCCCGACGTTTGGGGCTGATC	119 bp
Tlr5	GAATCCCGCTTGGGAGAACA	AGTGAGATGAGGCGTCTGGA	81 bp
Tnf-α	CCACCACGCTCTTCTGTCTAC	AGGGTCTGGGCCATAGAACT	103 bp
Trif	TAACACACCGCTGGACACTC	CACTGATGGAGGCCAGCTTA	85 bp

Cx3cr1, chemokine (C-X3-C motif) receptor 1*; F4/80,* adhesion G protein-coupled receptor E1 (Adgre1)*; Foxp3*, Forkhead box P3; *Il-10*, interleukin 10; *Itgαe*, Integrin α_E; *Muc2,* Mucin *2; Nod1,* nucleotide-binding oligomerization domain containing 1*; Nod2,* nucleotide-binding oligomerization domain containing 2*; Tff3,* Trefoil factor 3; *Tgf-β,* transforming growth factor β; *Tlr5*, Toll-like receptor 5; *Tnf-α,* tumor necrosis factor α; *Trif*, Toll-like receptor adaptor molecule 1.

Supplementary Results

Supplementary Table 3. Effects of antibiotics on the fecal microbiota composition at family level.

Phylum	Family	CTL[1] (%)	COL (%)	q
**Bacteroidetes**	Bacteroidaceae	15.3 ± 2.2	31.8 ± 2.6	**0.0003**
	Porphyromonadaceae	6.42 ± 0.40	7.94 ± 1.27	NS
	Sphingobacteriaceae	4.15 ± 0.42	4.10 ± 0.39	NS
	Flavobacteriaceae	3.63 ± 0.59	3.35 ± 0.51	NS
	Odoribacteriaceae	0.48 ± 0.09	1.73 ± 0.45	**0.0036**
	Prevotellaceae	0.36 ± 0.14	0.37 ± 0.12	NS
**Firmicutes**	Lachnospiraceae	12.1 ± 0.92	9.11 ± 1.03	NS
	Clostridiaceae	5.18 ± 0.64	5.25 ± 0.61	NS
	Lactobacillaceae	2.61 ± 0.63	1.37 ± 0.49	NS
	Erysipelotrichaceae	1.94 ± 0.50	3.25 ± 0.96	NS
	Ruminococcaceae	2.88 ± 0.50	4.16 ± 0.51	NS
	Eubacteriaceae	0.45 ± 0.11	5.05 ± 1.07	**0.0040**
**Proteobacteria**	Alcaligenaceae	0.96 ± 0.29	0.03 ± 0.003	**0.0003**
	Desulfovibrionaceae	0.87 ± 0.23	0.09 ± 0.02	**0.0003**
**Verrucomicrobia**	Verrucomicrobiaceae	19.0 ± 1.78	1.19 ± 1.10	**0.0003**
**Actinobacteria**	Bifidobacteriaceae	12.4 ± 2.57	10.2 ± 1.93	NS
**Tenericutes**	Entomoplasmataceae	2.05 ± 0.66	0.14 ± 0.05	**0.0282**
**Other**	Other (up to 129)	9.22	10.87	--

[1]CTL: control mice; COL: mice treated with Coliphur (daily dose: 25 mg/kg neomycin and 10 mg/kg colistin) for 14 days. Results are expressed as mean ± SEM (n= 9-10 mice). Statistical differences were considered significant at $q<0.05$ (corrected p values).

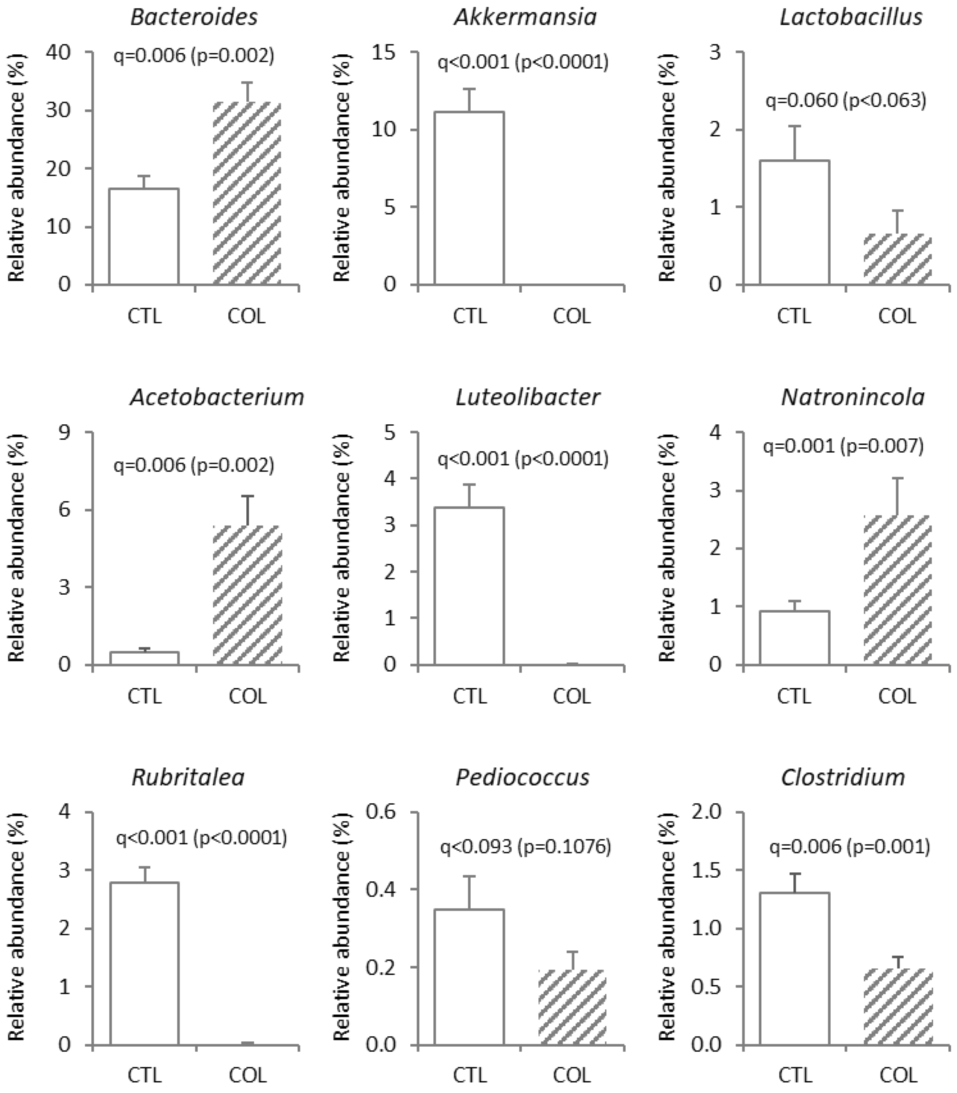

Supplementary Fig. 1. Effects of Coliphur on fecal microbial composition at genus level. CTL: control mice; COL: mice treated with Coliphur (daily dose: 25 mg/kg neomycin and 10 mg/kg colistin) for 14 days. Results are expressed as percent of the total population at this taxonomic level (n= 9-10 mice). Statistical differences were considered significant at q<0.05 (corrected p values).

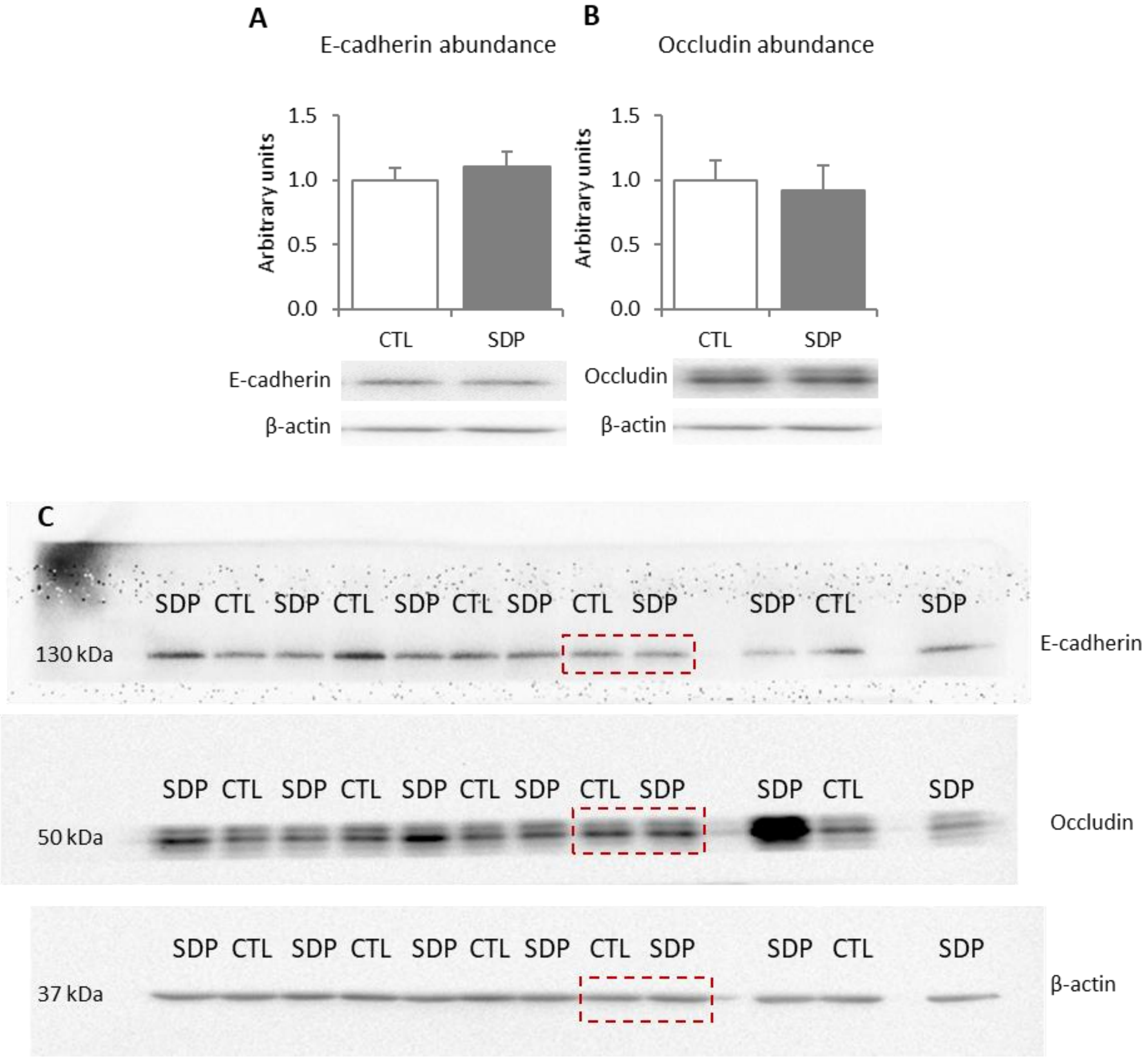

Supplementary Fig. 2. E-cadherin (A) and occludin abundance (B) in the colon mucosa. Mice were fed a control diet (CTL) or a diet supplemented with 8% spray-dried plasma (SDP) for 14 days. Primary antibodies against β-actin (clone AC-74; 1:80,000; Sigma Aldrich, St. Louis, MO, USA), occludin (clone OC-3F10, Life Technologies, Rockford, IL, USA) and E-cadherin (clone H-108, 1:500; Santa Cruz Biotechnology, Dallas, TX, USA) were used. Results are expressed as mean ± SEM (n= 5-6 mice). Representative images of western blots are shown below the graphs of protein abundance. Panel C show full length blots.
